# Severe Varicella Pneumonia in an Immunocompetent Adult Requiring Intensive Care Management: A Case Report

**DOI:** 10.7759/cureus.103763

**Published:** 2026-02-17

**Authors:** Manal El Goubi, Mohmmed Moussaoui, Khalid K Khaleq

**Affiliations:** 1 Critical Care and Anesthesiology, Hôpital Cheikh Khalifa Ibn Zaid, Casablanca, MAR; 2 Critical Care and Anesthesiology, Mohammed VI University of Health Sciences (UM6SS), Casablanca, MAR; 3 Critical Care and Anesthesiology, Ibn Rochd University Hospital, Casablanca, MAR

**Keywords:** adult infection, case report, intensive care, varicella pneumonia, viral pneumonia

## Abstract

Varicella is usually a mild childhood disease; however, primary infection in adults may lead to severe complications, particularly varicella pneumonia. We report the case of a 32-year-old previously healthy male admitted to the intensive care unit with acute hypoxemic respiratory failure occurring three days after the onset of a diffuse vesicular rash. Chest CT demonstrated bilateral ground-glass opacities with focal consolidation consistent with viral pneumonia. The diagnosis was established based on typical cutaneous findings, epidemiological exposure, and compatible imaging results. Early treatment with intravenous acyclovir followed by oral valacyclovir resulted in favorable clinical and radiological outcomes. This case emphasizes the importance of early recognition and prompt antiviral therapy to improve prognosis, even in immunocompetent adults without classical risk factors.

## Introduction

Varicella is an acute infectious disease caused by primary infection with the varicella-zoster virus. Although commonly regarded as a self-limited childhood illness, primary infection in adults is associated with significantly higher morbidity and complication rates [1]. Despite widespread vaccination programs in many regions, adult primary infection continues to occur, particularly in areas with incomplete immunization coverage.

Among adult complications, pulmonary involvement represents the most serious manifestation and remains a major cause of varicella-related mortality [2]. The incidence of varicella pneumonia in adults has been reported to range between 5% and 15%, with higher rates observed among hospitalized patients [3]. In severe cases complicated by respiratory failure and acute respiratory distress syndrome, mortality may reach up to 30% [4].

Adult varicella may also be associated with secondary bacterial skin infections, hepatitis, encephalitis, cerebellitis, myocarditis, and hematologic abnormalities [5]. Pathophysiologically, pulmonary involvement results from viral replication within alveolar epithelial cells and a diffuse inflammatory response leading to interstitial pneumonitis [6]. Radiologically and clinically, patients typically present with fever, diffuse vesicular rash, cough, and progressive dyspnea developing within several days of rash onset [7].

Several case reports and series have described severe disease in immunocompetent adults, demonstrating that the absence of traditional risk factors does not preclude significant pulmonary involvement [8]. Early recognition of respiratory symptoms and prompt initiation of antiviral therapy are critical, as delayed treatment has been associated with worse clinical outcomes [9].

We report a case of severe varicella pneumonia in a previously healthy immunocompetent adult requiring intensive care management.

## Case presentation

A 32-year-old male with no significant past medical history presented with progressive dyspnea. He was a non-smoker and had no history of chronic pulmonary disease or immunosuppressive conditions. He reported no prior history of varicella during childhood.

Six days before admission, the patient developed a sudden high-grade fever associated with myalgia and arthralgia, suggestive of a viral prodrome. Two days later, a pruritic vesicular rash appeared initially on the thorax and face and subsequently spread centrifugally to the trunk and extremities, consistent with primary varicella infection. Three days after rash onset, he developed progressive dyspnea and dry cough, indicating possible pulmonary involvement. The temporal relationship between cutaneous eruption and respiratory deterioration was highly suggestive of varicella pneumonia, a known early complication of adult primary infection.

**Figure 1:**
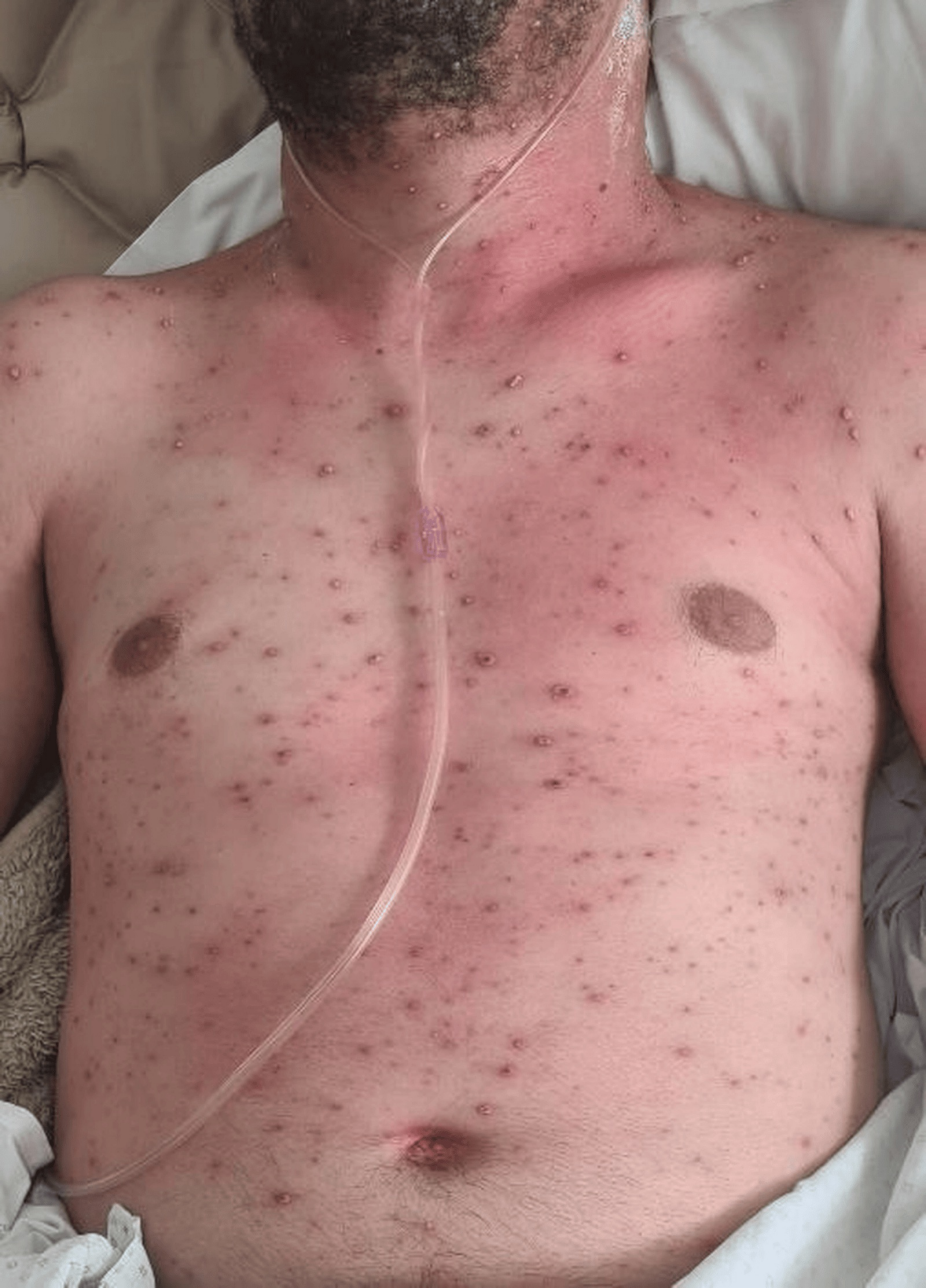
Diffuse vesicular rash involving the anterior thorax.

**Figure 2:**
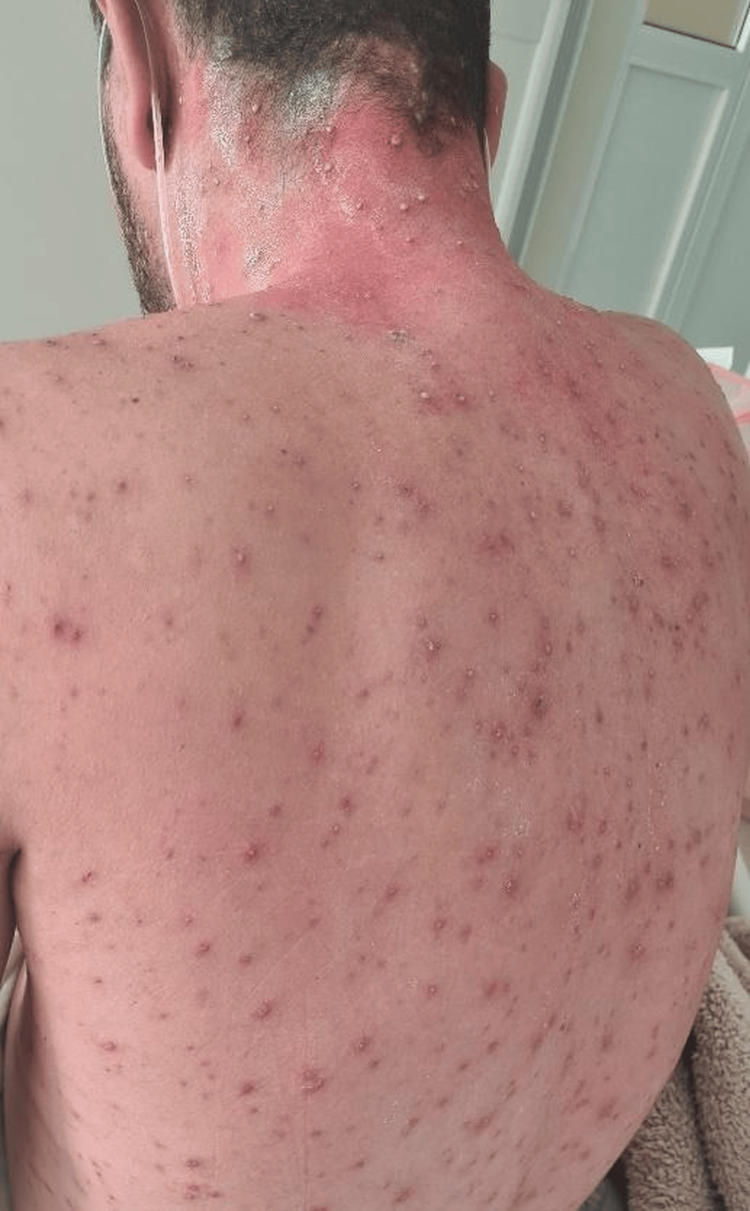
Posterior distribution of vesicular lesions over the back and shoulders.

On admission, temperature was 39.2°C, blood pressure was 150/70 mmHg, heart rate was 120 beats per minute, respiratory rate was 32 breaths per minute, and oxygen saturation was 80% on room air. Physical examination showed signs of respiratory distress with diffuse bilateral rhonchi. Skin examination revealed diffuse umbilicated vesicular lesions. The patient presented with diffuse umbilicated vesicular lesions over the anterior thorax (Figure 1). Similar vesicular lesions were observed on the posterior trunk and shoulders (Figure 2).

Laboratory evaluation demonstrated a C-reactive protein level of 33.78 mg/L (reference range: <5 mg/L), procalcitonin of 0.364 ng/mL (reference range: <0.05 ng/mL), and lymphopenia with a lymphocyte count of 420 cells/mm³ (reference range: 1,000-4,000 cells/mm³). Serum creatinine was 0.9 mg/dL (reference range: 0.6-1.3 mg/dL), and blood urea nitrogen was within normal limits, indicating preserved renal function. HIV serology and SARS‑CoV‑2 polymerase chain reaction (PCR) testing were negative. Varicella-zoster virus PCR from blood (or lesion swab) was positive, confirming the diagnosis.

**Figure 3:**
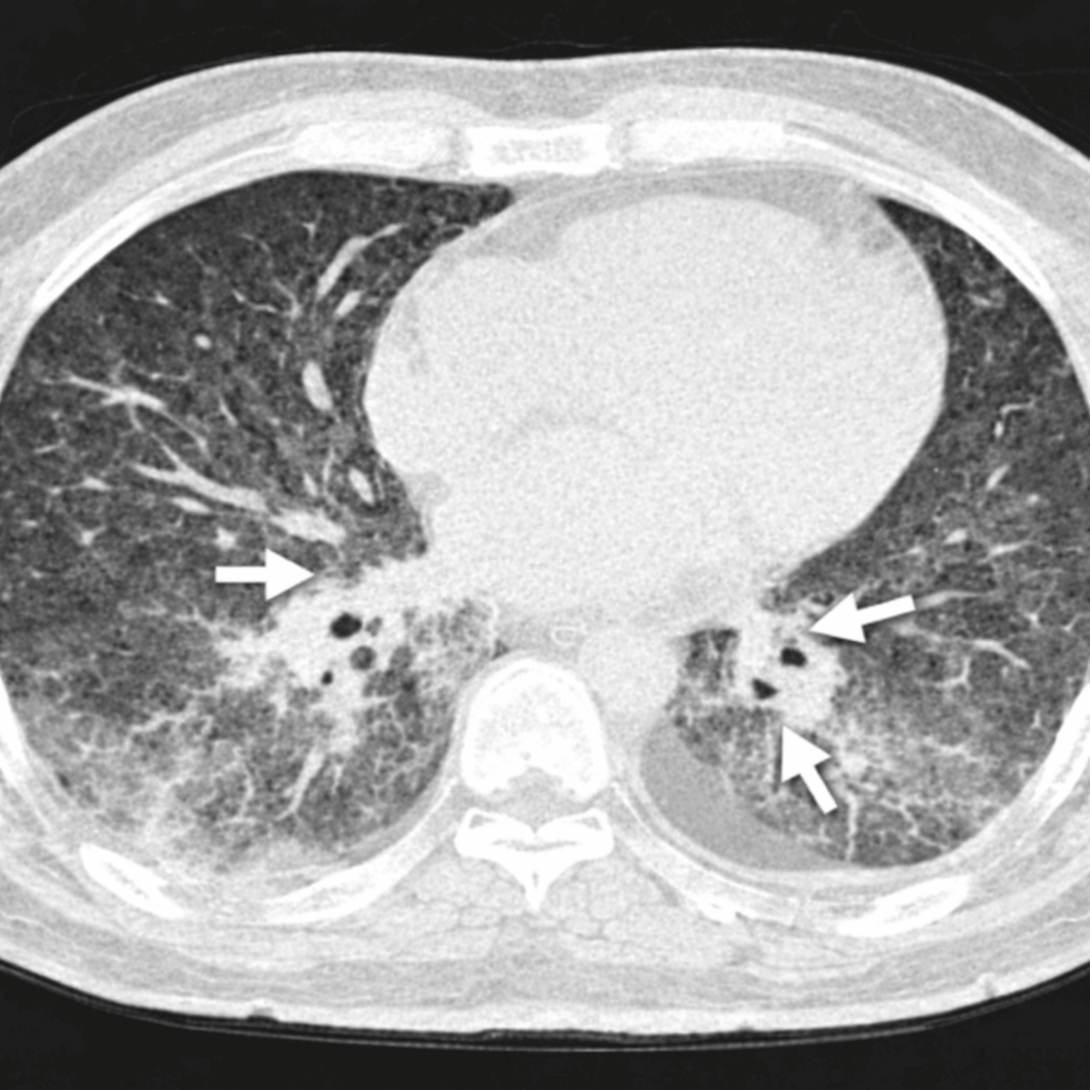
Chest CT showing bilateral posterior basal ground-glass opacities with areas of consolidation.

Given the severity of hypoxemia on presentation, a chest CT was performed directly to better assess the extent of pulmonary involvement. CT revealed bilateral posterior basal ground-glass opacities, more pronounced on the left, with focal consolidation and mild bilateral pleural effusion (Figure 3). These findings were consistent with viral pneumonia [5].

The patient was admitted to the intensive care unit for management of acute hypoxemic respiratory failure and started on high-flow oxygen therapy. Airborne and contact isolation precautions were implemented due to active varicella infection in accordance with institutional infection control protocols.

The diagnosis of varicella pneumonia was established based on the characteristic vesicular rash, recent exposure to a child with varicella, absence of prior infection history, compatible imaging findings, and confirmation by positive varicella-zoster virus PCR testing.

The patient received intravenous acyclovir at 10 mg/kg every eight hours, followed by oral valacyclovir 1 g three times daily for a total of 10 days. Ceftriaxone 2 g per day was administered for suspected secondary bacterial skin infection.

Clinical evolution was closely monitored in the intensive care unit. On hospital day one, the patient presented with acute hypoxemic respiratory failure with an oxygen saturation of 80% on room air. He was started on high-flow nasal oxygen therapy at 50 L/minute with a fraction of inspired oxygen (FiO₂) of 60%, achieving oxygen saturation above 92%. Intravenous acyclovir was initiated upon admission.

On hospital day two, respiratory distress persisted but remained stable under high-flow oxygen therapy, with FiO₂ gradually reduced to 50%. Close monitoring was maintained for potential progression to acute respiratory distress syndrome. Inflammatory markers began trending downward.

By hospital day three, clinical improvement was observed, with a reduction in dyspnea and oxygen requirements. High-flow oxygen was discontinued and replaced with a conventional nasal cannula at 4 L/minute. On hospital day four, oxygen flow was reduced to 2 L/minute with stable oxygen saturation. Cutaneous lesions progressively crusted, and systemic symptoms resolved. By hospital day five, the patient was weaned to room air with oxygen saturation maintained above 94%. He was transferred from the intensive care unit to the general ward on hospital day six and discharged without respiratory sequelae.

At the two-week follow-up, the patient reported complete resolution of respiratory symptoms. Oxygen saturation remained normal on room air, and no residual respiratory complaints were noted.

## Discussion

Varicella pneumonia remains the most severe complication of primary varicella infection in adults and continues to account for substantial morbidity and mortality in this population [2]. Although traditionally associated with immunosuppression, pregnancy, and smoking, severe pulmonary involvement has also been reported in immunocompetent adults [4]. The present case reinforces this observation, as our patient had no underlying comorbidities yet developed acute hypoxemic respiratory failure requiring intensive care admission.

Radiologically, varicella pneumonia most commonly manifests as bilateral ground-glass opacities and nodular infiltrates, frequently with basal predominance [5]. In more severe presentations, areas of consolidation and pleural effusion may be observed, reflecting extensive alveolar involvement. The CT findings in our patient closely corresponded to these established imaging patterns and supported the diagnosis.

Clinically, respiratory symptoms typically develop within one to six days following the onset of the characteristic vesicular rash [7]. In this case, dyspnea emerged three days after rash appearance, consistent with previously described disease timelines. Recognition of this temporal progression is essential, as early suspicion facilitates timely diagnostic evaluation and therapeutic intervention.

Management relies on supportive respiratory care and prompt antiviral therapy. Intravenous acyclovir remains the treatment of choice, and early administration has been shown to significantly reduce morbidity and mortality [9]. In contrast, untreated or late-treated cases may progress to severe respiratory failure, acute respiratory distress syndrome, and mechanical ventilation. Mortality rates in critically ill patients have historically reached up to 30% [4]. In our case, early antiviral therapy and intensive monitoring were associated with rapid clinical improvement and complete recovery without sequelae.

This case highlights several clinically relevant considerations. Severe pulmonary involvement may occur even in immunocompetent individuals without traditional risk factors. The predictable temporal relationship between rash and respiratory compromise remains a key diagnostic clue. Imaging findings are consistent with previously reported patterns, and early antiviral therapy significantly influences clinical outcomes.

The strength of this report lies in the comprehensive documentation of symptom chronology, detailed radiological evaluation, intensive care management, and molecular confirmation of infection in an immunocompetent adult. As a single-case report, the findings cannot be generalized. Additionally, long-term pulmonary function or radiological follow-up was not available. Nevertheless, this case reinforces the importance of early recognition and prompt treatment in adult primary varicella infection.

## Conclusions

Varicella pneumonia remains a potentially life-threatening complication of primary varicella infection in adults, including immunocompetent individuals without traditional risk factors. Rapid progression from cutaneous eruption to respiratory compromise should prompt early clinical suspicion and immediate evaluation for pulmonary involvement. This case underscores the importance of recognizing the characteristic temporal pattern of symptom evolution, performing timely radiological assessment, and initiating prompt antiviral therapy combined with appropriate respiratory support. Early intervention and close monitoring, particularly in patients presenting with hypoxemia, are critical to preventing progression to severe respiratory failure and improving clinical outcomes. Adult primary varicella infection should therefore not be considered a benign condition, and clinicians should maintain a high index of suspicion when respiratory symptoms emerge.

## References

[1] Heininger U, Seward JF (2006). Varicella. Lancet.

[2] Mohsen AH, McKendrick M (2003). Varicella pneumonia in adults. Eur Respir J.

[3] Tunbridge AJ, Breuer J, Jeffery KJ (2008). Chickenpox in adults - clinical management. J Infect.

[4] Mirouse A, Vignon P, Piron P (2017). Severe varicella-zoster virus pneumonia: a multicenter cohort study. Crit Care.

[5] Singh A, Parkash S, Gupta SK, Soni RK (2018). Severe varicella pneumonia in adults: seven years' single-center experience from India. Indian J Crit Care Med.

[6] Koo HJ, Lim S, Choe J, Choi SH, Sung H, Do KH (2018). Radiographic and CT features of viral pneumonia. Radiographics.

[7] Denny JT, Rocke ZM, McRae VA (2018). Varicella pneumonia: case report and review of a potentially lethal complication of a common disease. J Investig Med High Impact Case Rep.

[8] Aabdi M, Hamza M, Moussa L, Houssam B, Brahim H (2021). Acute respiratory distress syndrome caused by varicella pneumonia in immunocompetent adult: clinical case. Ann Med Surg (Lond).

[9] Haake DA, Zakowski PC, Haake DL, Bryson YJ (1990). Early treatment with acyclovir for varicella pneumonia in otherwise healthy adults: retrospective controlled study and review. Rev Infect Dis.

